# Mr. Carnegie and the Scottish Universities

**Published:** 1901-06-15

**Authors:** 


### The Hospital

A Journal of

The Medical Sciences and Hospital Administration.

Vol. XXX.—No. 768] Edited by Sir Henry Burdett, K.C.B., and Solomon C. Smith, M.D., M.R.C.P. [June 15, 1901.

### Mr. Carnegie and the Scottish Universities.

The munificence of Mr. Carnegie has cast upon the authorities of the Scottish Universities the burden of a great responsibility. Their position has been altered, if not from one of actual struggle to one of "wealth beyond the dreams of avarice," at least to one sufficient for the initiation and accomplishment of great educational undertakings, from which, at no distant date, some substantial fruits will reasonably be expected by the country. The trustees of the gift will have at their disposal an income of about fifty thousand a year available for the improvement or the construction of laboratories and for the endowment or prosecution of research, and there can be little doubt that, in their allotment of this income among the various institutions which may properly claim some share in it, they will be largely guided by any available evidence of a disposition to expend money with energy and wisdom. The great want of English academic institutions in the past has been due to the absence of any means of supporting those who were engaged in scientific inquiries of a kind which were not immediately remunerative, and this want the Carnegie trust will to a large extent be able to supply. Something of the kind was manifestly intended by the Fellowships of the older English Universities; regard being had to the fact that these were founded at periods when research was unknown and unthought of, and when "learning" was understood to mean a familiar, or even an exhaustive knowledge of the writings and opinions of the scholars of former days. The idea that its province was to enrich the mind of man by the discovery and establishment of previously unknown truth had not yet dawned upon even the most advanced of the so-called philosophers; and one result of the view then taken of the subject was that the requirements of the philosopher were supposed to be small and easily satisfied, and that the endowments for supplying them were exiguous in proportion to this estimate. It was, unquestionably, possible to cultivate literature upon a little oatmeal, but such slender provision was wholly inadequate to the reasonable demands of those who would cultivate science. In the first place, scientific research is itself costly, requiring instruments and apparatus of extreme delicacy and precision; and, in the next place, those who are able to conduct it are sorely tempted to do so for the immediate profit of themselves or others. Even among physicists there are not many who would re-echo the words of the great man who, according to Tyndall, astonished the American capitalist who wished to tempt him from his studies by saying that he "had no time to waste in making money." Anyone who is able to conduct scientific research, or to teach others to conduct it, has now so considerable a market value in the eyes of manufacturers as to render it increasingly difficult to retain him in a professorial capacity, or to induce him to abandon, for the sake of pure truth, prospects which he may reasonably think it proper to cultivate, if not for his own sake, at least for that of his children or of others connected with him. To render professors independent is the first step towards placing science itself in the position which she ought to occupy, the position of being the guide of commerce and of manufacture, but no longer their handmaid.

With regard to the remainder of the income of the gift, the second fifty thousand per annum, we should be inclined to doubt whether there will be fitting claimants to absorb even a large proportion of it, and whether it will not, at least after a time, come to be applied, in the full discretion which is given to the trustees, to some other purpose than that primarily set forth by the donor. The sturdy spirit of Scottish independence will not be likely to take kindly to the acceptance of the mere fees for University classes by students whose families are able to provide for their maintenance and to meet the other expenses contingent upon their education; and the help thus afforded would not indeed be sufficient, in other than exceptional cases, to make any very considerable difference to the persons concerned. The power given to the Committee of the fund to provide entirely for a sufficiently deserving student, that is to say, to give him whatever money or privileges his circumstances may render necessary, so as to maintain, if need be, as well as to educate him, is one the value and importance of which it would be impossible to exaggerate; assuming only—and we fear it is a somewhat large assumption—that this power is never exercised save in the case of young people with whom the necessity is real, and whose abilities and industry are such as to render it practically certain that in due course the seed time will be followed by harvest. It is to be hoped that the Committee will not, either now or at any future time, think that the exercise of this power is called for in any but exceptional circumstances.

The demands of these, together with those of students who legitimately seek to be relieved of mere class expenses, will, we should imagine, leave a considerable surplus; and this, by the terms of the trust, may be expended on evening or continuation schools, or in many other ways which would facilitate the transfer of fitting pupils from elementary to secondary classes, and thus prepare for the University many who might not otherwise become qualified to reach its portals. But, whatever the direction of expenditure, the important fact in the case is that the provisions made by the munificent donor of the fund have been dictated by a sagacity as conspicuous as his liberality; and that it rests mainly with the Universities themselves either to utilise this liberality for the lasting advantage of the human race, or to permit the resources which it has supplied to be misdirected by nepotism or wasted by carelessness. The burden thrown upon them is a weighty one, and we can only trust that all concerned will be earnest in their endeavour to bear it in such a manner that their names may be remembered as those of the wise stewards of a magnificent inheritance.

